# Early childhood weight gain and alanine aminotransferase at age 8: an adjunct study of the Japan Environment and Children’s Study

**DOI:** 10.1186/s12916-026-04734-x

**Published:** 2026-03-03

**Authors:** Naw Awn J-P, Keiko Yamasaki, Naomi Mitsuda, Masamitsu Eitoku, Ryuhei Nagai, Mariko Araki, Mariko Taniguchi-Ikeda, Narufumi Suganuma

**Affiliations:** 1 Department of Environmental Medicine, Kochi Medical School, Kochi University, Nankoku, Kochi 783–8505 Japan; 2 Center for Child Health and Environmental Medicine, Kochi University, Nankoku, Japan; 3 Department of Pediatrics, Kochi Medical School, Kochi University, Nankoku, Japan; 4 Department of Obstetrics and Gynecology, Kochi Medical School, Kochi University, Nankoku, Japan

**Keywords:** Adiposity rebound, BMI, Childhood growth, Conditional weight, Fatty liver, Liver enzyme, Longitudinal cohort study, MASLD, Obesity, Pediatric

## Abstract

**Background:**

The critical age window during which early-life adiposity impacts liver health remains unclear. This study aimed to identify the timing of adiposity gain associated with elevated alanine aminotransferase (ALT) levels in 8-year-old children.

**Methods:**

This prospective cohort study included 1322 children (665 boys; mean age 96.2 ± 3.4 months) from a subset of the Japan Environment and Children’s Study. Anthropometric data were collected at birth and at 1, 2, 3, 4, 5, 6, and 8 years. Adiposity gain was assessed using conditional weight, a residual-based metric adjusted for prior weight and current height. Excess adiposity was defined as conditional weight above the 90th percentile. ALT was measured at age 8, with elevation defined as > 26 IU/L in boys and > 22 IU/L in girls. Multivariable regression models were adjusted for maternal, perinatal, and early-life factors.

**Results:**

ALT elevation was observed in 3.3% of the children. Adiposity gain was significantly associated with higher ALT concentrations, beginning at age 3 in girls (adjusted coefficient: 0.11, *p* < 0.01) and at age 4 in boys (adjusted coefficient: 0.10, *p* < 0.05). The 4–5-year interval marked the earliest period of notable risk, with adjusted risk ratios (95% CI) of 4.18 (1.77–9.87) in boys and 3.29 (1.04–10.40) in girls. Birth weight and adiposity during infancy were not consistently associated with ALT concentrations.

**Conclusions:**

Early childhood—particularly between ages 3 and 5 years—may represent a period during which associations between excess adiposity gain and later liver health become detectable. Because liver enzymes were assessed at a single time point, the temporal onset of these associations cannot be established. These findings should be interpreted cautiously and warrant confirmation using longitudinal assessments of liver health.

**Graphical Abstract:**

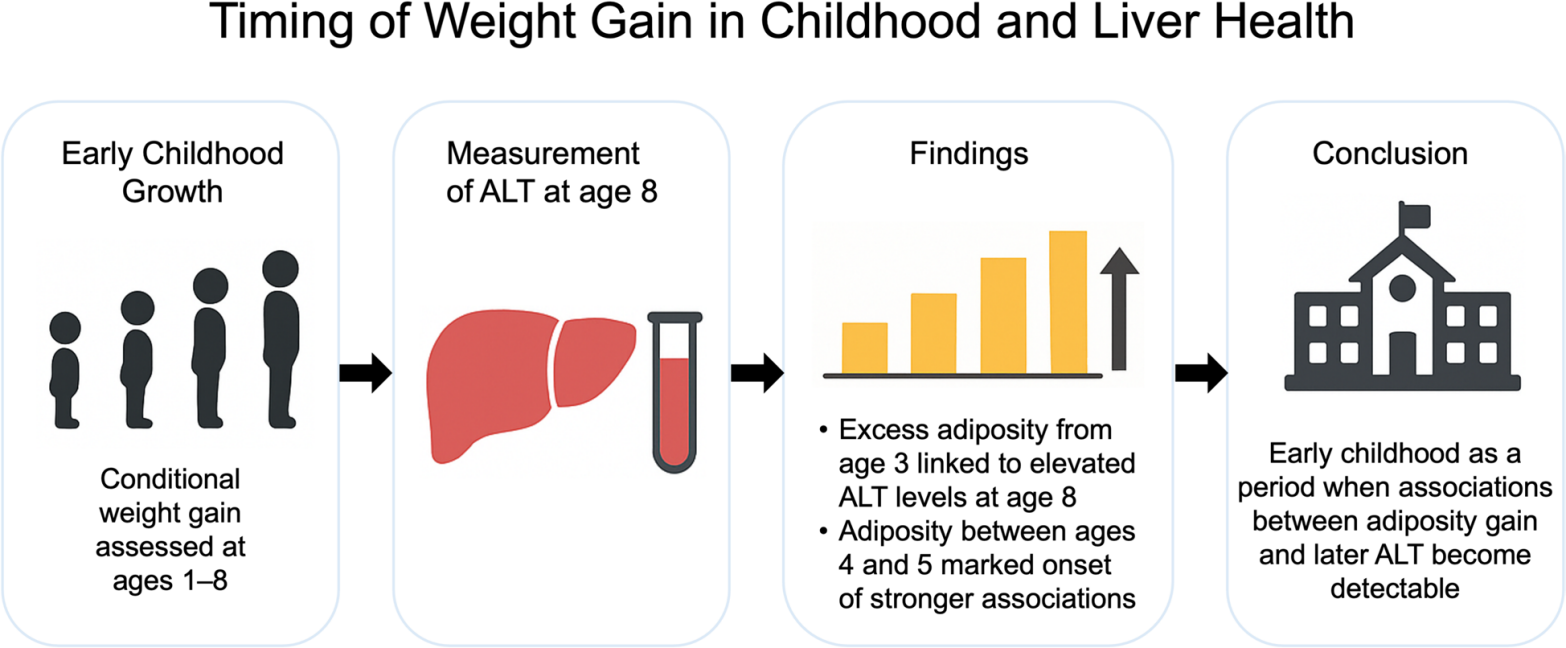

**Supplementary Information:**

The online version contains supplementary material available at 10.1186/s12916-026-04734-x.

## Background

Childhood is a period of rapid growth, characterized by significant increases in both lean body mass and fat mass [1, 2]. Research has shown that excessive weight gain during childhood is a significant risk factor for the development of metabolic dysfunction-associated steatotic liver disease (MASLD), previously known as non-alcoholic fatty liver disease [3–5]. However, the specific age at which adiposity begins to impact liver health in children remains unclear. Identifying this critical period could improve our understanding of when to initiate appropriate interventions, such as lifestyle modifications.

To date, no studies have prospectively assessed the age at which this association emerges. Only two studies have retrospectively compared children with and without MASLD, reporting that body mass index (BMI) trajectories diverge around ages 2 to 3 years [3, 5]. However, these findings are primarily based on changes in BMI. Although BMI accounts for weight relative to height, it does not distinguish between fat mass and lean mass and may therefore be confounded by variations in skeletal muscle growth during early childhood. Consequently, population studies aiming to examine the relationship between adiposity and health outcomes should adjust for height-related influences on body weight. Furthermore, to more accurately determine the temporal association between weight gain and health outcomes, it is important to use metrics that are independent of prior weight status. In this regard, conditional weight—which statistically adjusts for current height and previous weight—may provide a more precise indicator of adiposity relevant to health risk [6].

In addition, suboptimal conditions during intrauterine life may impair fetal liver development and contribute to MASLD risk. However, evidence on the relationship between prenatal growth and liver health in children remains inconsistent. Some studies have found that fetal growth restriction increases the risk of MASLD [7, 8], whereas others have not identified such an association [5, 9].

To address this knowledge gap and the limitations of previous studies, the primary objective of the present study was to explore the developmental stage at which associations between adiposity gain and liver health become detectable in children. Alanine aminotransferase (ALT) is an enzyme predominantly localized in hepatocytes, and elevated serum levels are typically indicative of hepatocellular injury [10]. MASLD is among the most common causes of elevated ALT in children [11]. Accordingly, several pediatric and medical societies recommend ALT screening for MASLD in children with obesity [12]. In this prospective study, we investigated the associations of birth weight—as a proxy for prenatal growth—and annual conditional weight measurements with serum ALT concentrations at age 8 years, in a cohort of apparently healthy Japanese children enrolled in the Japan Environment and Children’s Study (JECS).

## Methods

This article followed the STrengthening the Reporting of OBservational studies in Epidemiology (STROBE) Statement checklist for cohort studies.

### Study design and participants

This prospective study was conducted at the Kochi Regional Center as an adjunct to the JECS, an ongoing national birth cohort study that enrolled ~ 100,000 pregnant women across Japan between January 2011 and March 2014 [13]. The primary aim of the JECS is to investigate the environmental influences on children’s health. Although the JECS primarily involves biannual questionnaire surveys after childbirth, a face-to-face survey was conducted at age 8 years. During this survey, the Kochi Regional Center performed a medical examination, including peripheral blood sampling, for children whose parents provided informed consent. The JECS protocol was reviewed and approved by the Ministry of the Environment’s Institutional Review Board on Epidemiological Studies (No. 100910001) and the ethics committees of all participating institutions. Written informed consent was obtained from all participants. This adjunct study was additionally approved by the Institutional Review Board of Kochi Medical School (No. 30–167).

**Fig. 1:**
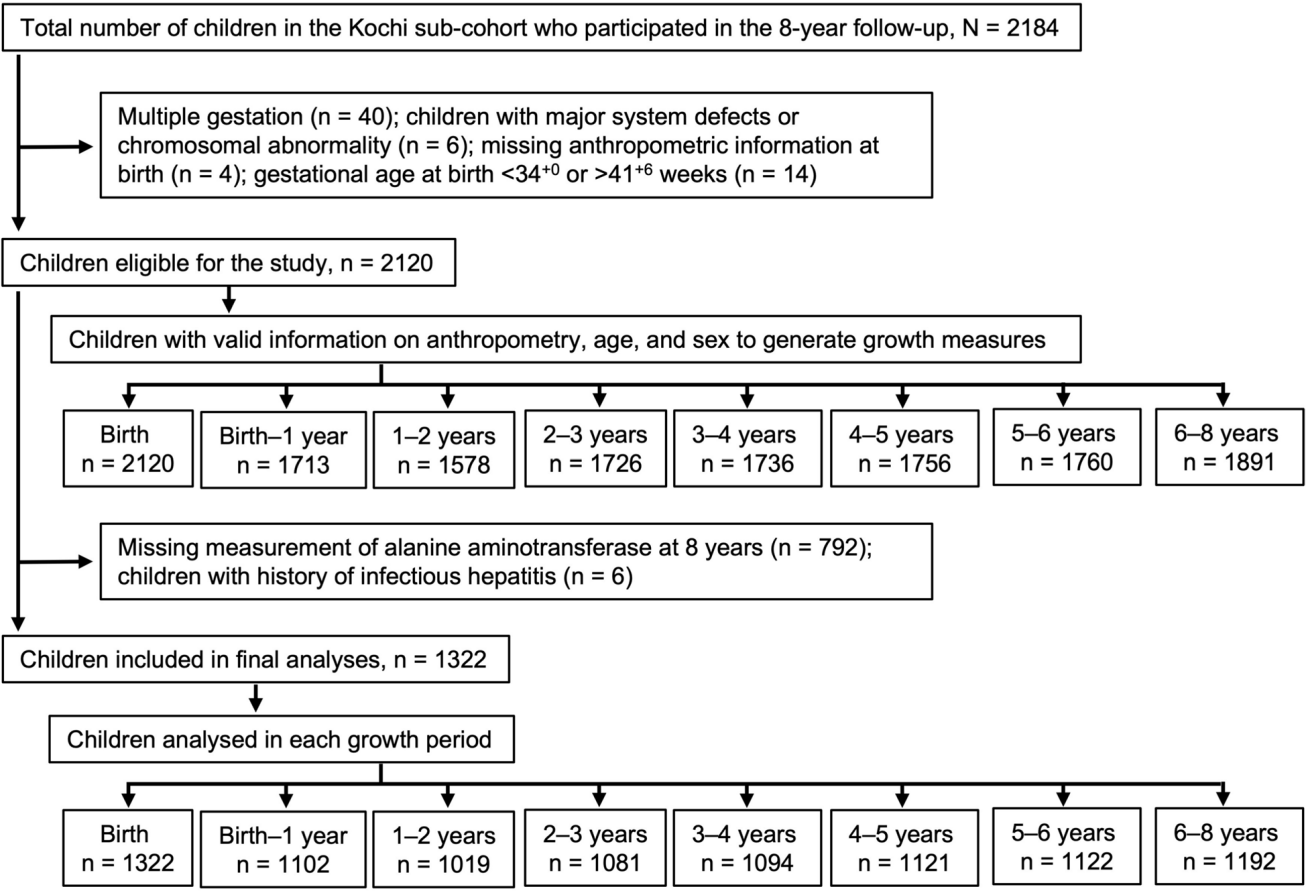
Participant selection

Of the 7143 fetuses initially registered at the Kochi Regional Center, 6889 were born alive, representing ~ 52% of all live births in the study area during the recruitment period. A total of 2184 children participated in the 8-year follow-up conducted between 2019 and 2022. Of these, 2120 singleton births between 34^+0^ and 41^+6^ weeks of gestation without major anomalies were eligible. After excluding 798 children due to a history of infectious hepatitis or missing ALT measurements at age 8, 1322 children remained for the final analysis (Fig. 1).

### Adiposity measures

Height/length and weight measurements were collected at birth and at ages 1, 2, 3, 4, 5, 6, and 8. Birth anthropometrics and gestational age were extracted from medical records, with gestational age determined by first-trimester ultrasound or date of last menstrual period. For ages 1 to 6 years, height and weight (along with the corresponding date of measurement) were reported by caregivers via questionnaire, referencing data from routine well-child visits or preschool/school health checkups. Measurements were excluded if the child’s age at the time of assessment deviated by more than ± 6 months from the target age. At the 8-year follow-up, trained medical personnel measured height and weight under standardized conditions, with children wearing light clothing and no shoes.

Age- and sex-specific z-scores for height, weight, and BMI were calculated using World Health Organization (WHO) growth standards [14]. Z-scores with absolute values ≥ 5 were removed as outliers. Birth weight z-scores served as proxies for intrauterine growth. Conditional weight was calculated at ages 1, 2, 3, 4, 5, 6, and 8 to assess childhood adiposity. To minimize bias associated with missing ALT data, conditional weight variables were derived using all available anthropometric data. Conditional weight was computed by regressing current weight on current height and weight at the preceding time point, with separate models for each sex. The resulting residuals captured deviations in weight gain beyond what would be expected. For example, a positive conditional weight at age 1 indicates that the child weighed more than expected given their height, birth weight, and typical growth trajectories, reflecting an accelerated gain in adiposity from birth to age 1.

### Alanine aminotransferase measurements

At the 8-year follow-up, 62.4% (1322/2120) of eligible children provided non-fasting blood samples. Serum ALT, aspartate aminotransferase (AST), and γ-glutamyl transferase (GGT) were assayed using standardized laboratory protocols.

### Covariates and potential confounders

These included maternal characteristics (age at recruitment, parity, marital status, pre-pregnancy BMI, household income, education, tobacco and alcohol use, anemia during pregnancy, hypertension [pre-existing or pregnancy-induced], and diabetes mellitus [pre-existing or gestational]), as well as perinatal and postnatal variables (gestational age at birth, duration of neonatal hospitalization, breastfeeding duration, nutritional intake, chronic illness, pubertal development, and attendance at a childcare facility).

Nutritional intake at 4.5 years was assessed using the caregiver-reported Brief Self-Administered Diet History Questionnaire (BDHQ). Daily intake of carbohydrates, fat, protein, and added sugar (from foods and beverages) was expressed as a percentage of total daily energy (% energy) to account for differences in total energy intake over time. The presence of chronic major systemic or organ disorders was determined based on caregiver-reported physician diagnoses at each questionnaire wave. Because this large-scale epidemiological study could not feasibly or ethically conduct physical examinations to assess pubertal status, we relied on caregiver reports of breast development at age 6 years as an indicator of early pubertal signs. No cases suggestive of precocious puberty were reported. Children’s behaviors at ages 3 and 4—such as participation in sports (as a proxy for physical activity) and time spent playing with mobile phones or gaming consoles (as a proxy for sedentary time)—were also assessed. These variables were selected based on existing literature and their relevance as antecedents of both the exposure and outcome [9, 15–19]. Data were collected through standardized parent- or caregiver-completed questionnaires and medical record transcripts.

### Statistical analyses

ALT values were log-transformed to account for right-skewed distribution. Although AST and GGT were also measured to complement the assessment of liver function, both markers showed limited variability in this cohort of healthy children. Because ALT is considered the most sensitive indicator of adiposity-related liver injury in pediatric populations, the primary analyses focused on ALT.

Descriptive statistics (mean ± SD or median [IQR, interquartile range] and proportion) were used to summarize cohort characteristics. Between-group comparisons used the Student’s *t*-test for continuous variables and chi-squared test for categorical variables. A two-tailed *p*-value of < 0.05 was considered statistically significant. Analyses were performed using StataNow/MP 19.5 software (StataCorp, College Station, TX).

**Fig. 2:**
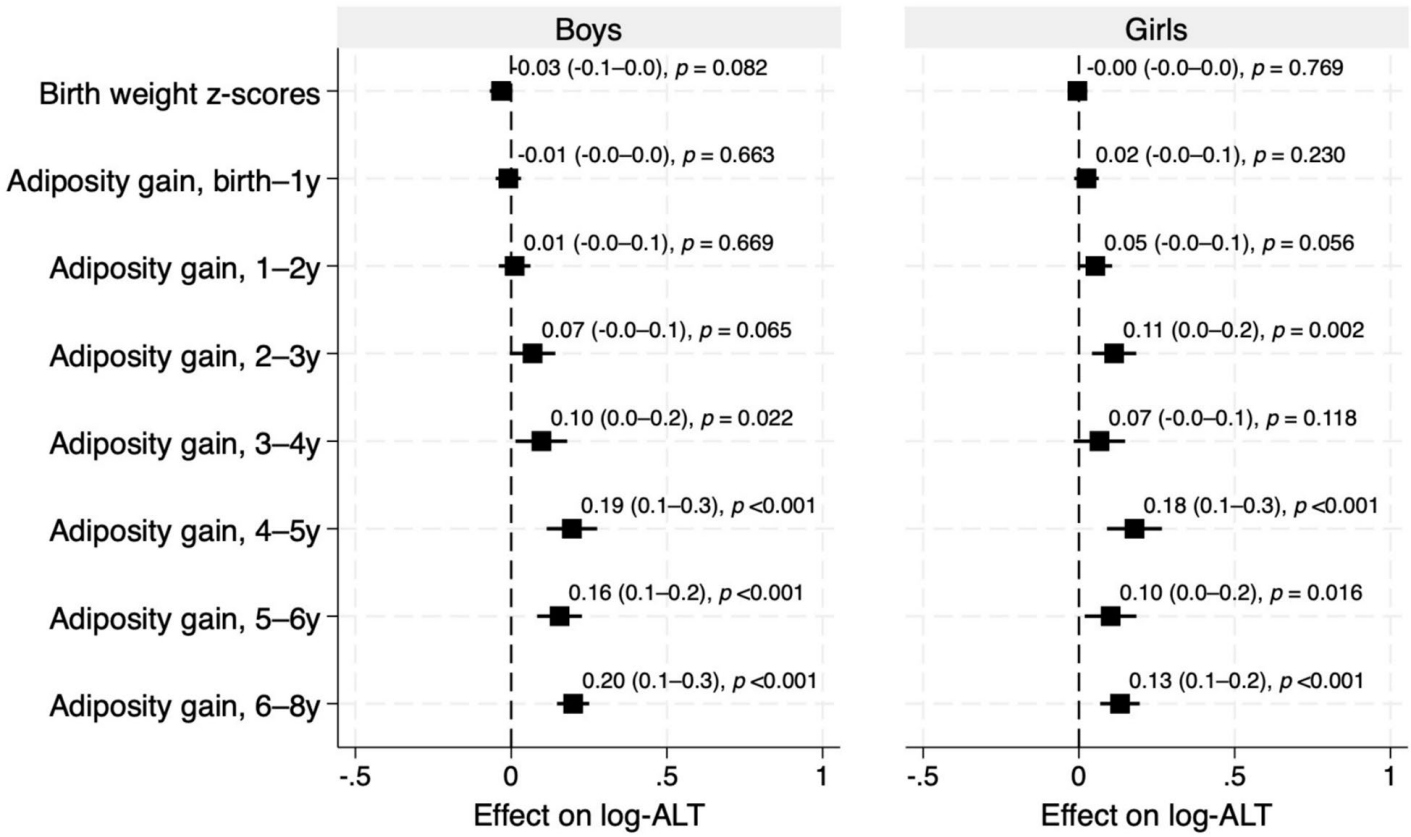
Association between timing of adiposity and log-transformed ALT (log-ALT) levels at age 8. Values represent regression coefficients (95% confidence intervals) and corresponding *p*-values from linear regression models. All models were adjusted for maternal pre-pregnancy BMI, annual household income, maternal educational level, smoking and alcohol consumption during pregnancy, anemia during pregnancy, gestational age at birth (weeks), and the child’s age at ALT measurement (months). For models evaluating adiposity gain up to 3 years, additionally adjusted for neonatal hospitalization and breastfeeding duration. For models assessing adiposity gain at age 4 years, additional adjustments included participation in sports and screen time. For models assessing adiposity gain at age 5 and later, we further adjusted for nutritional intake at 4.5 years. ALT, alanine aminotransferase

All available anthropometric data were used (Fig. 1). We first assessed the relationship between adiposity measures at various time points and ALT levels at age 8 using Pearson correlation and crude and adjusted linear regression analyses. Time points showing significant relationships with ALT informed further analysis (Additional file 1: Table S1 and Fig. 2). Additional analyses investigated the impact of excess adiposity on ALT elevation. Excess adiposity was defined as conditional weight > 90th percentile of the population distribution. Elevated ALT was defined as a blood concentration > 26 IU/L in boys and > 22 IU/L in girls [20]. Associations between excess adiposity and elevated ALT status were evaluated using chi-squared tests. Poisson regression with robust error variance was used to estimate the risk ratios (RRs) and 95% confidence intervals (CIs), referencing the average adiposity group. Analyses were stratified by sex and adjusted for the child’s age at ALT measurement and a range of relevant confounders. These included maternal pre-pregnancy BMI (categorized as < 18.5, 18.5–24.9, ≥ 25 kg/m^2^), highest educational attainment (high school or higher [vocational school, college, university, or beyond]), annual household income (< 4 million or ≥ 4 million Japanese yen), maternal smoking and alcohol consumption, anemia during pregnancy, gestational age at birth (in weeks), duration of neonatal hospitalization (≤ 7 or > 7 days), breastfeeding duration (≤ 6 or > 6 months), the child’s participation in sports (yes or no), time spent playing with electronic devices (< 1 h or ≥ 1 h per day), and nutritional intake (total daily energy and the percentage of energy derived from added sugar). Maternal hypertension and diabetes were not included due to limited cases (Table 1). To assess the robustness of the findings, association analyses were repeated under two conditions: (1) restricting the sample to 929 children with complete conditional weight data at ages 3, 4, 5, 6, and 8 years; and (2) limiting anthropometric measurements to within ± 3 months of each target age.

**Table 1:** Baseline characteristics of participants

Characteristics	Missing	Boys	Girls	*p*
	***n* (%)**	***n* = 665**	***n* = 657**	
**Maternal characteristics**				
Age ≥ 35 years	0	185 (27.8)	200 (30.4)	0.294
Parity 1 +	64 (4.8)	356 (56.2)	372 (59.6)	0.213
Marital status, married	32 (2.4)	638 (98.3)	632 (98.6)	0.672
Pre-pregnancy BMI	1 (0.1)			0.980
< 18.5 kg/m^2^		98 (14.7)	96 (14.6)	
18.5–24.9 kg/m^2^		497 (74.7)	493 (75.2)	
≥ 25 kg/m^2^		70 (10.5)	67 (10.2)	
Highest educational attainment, high school or less	8 (0.6)	169 (25.5)	148 (22.7)	0.231
Annual household income, < 4 million Japanese yen	53 (4.0)	259 (40.9)	244 (38.4)	0.353
Smoking status, ever-smoker	8 (0.6)	224 (33.9)	216 (33.0)	0.726
Alcohol consumption	8 (0.6)			0.755
Never drink		217 (32.9)	207 (31.6)	
Quit during pregnancy		369 (56.0)	380 (58.0)	
Current drinking		73 (11.1)	68 (10.4)	
Presence of anemia	5 (0.4)	135 (20.4)	132 (20.1)	0.892
Presence of hypertension^a^		18 (2.7)	27 (4.1)	0.160
Presence of diabetes^b^		22 (3.3)	23 (3.5)	0.847
**Child’s characteristics**				
Preterm birth	0	29 (4.4)	21 (3.2)	0.267
Neonatal hospital stays > 7 days	2 (0.2)	172 (25.9)	135 (20.6)	0.022
Breastfeeding duration > 6 months	0	531 (79.9)	525 (79.9)	0.979
Daycare or kindergarten enrollment				0.108
Yes		616 (92.6)	613 (93.3)	
No		3 (0.5)	9 (1.4)	
Not reported		46 (6.9)	35 (5.3)	
Screen time at age 3 years,^c^ hours per day	54 (4.1)			0.038
0 h		338 (52.8)	370 (58.9)	
< 1 h		246 (38.4)	221 (35.2)	
≥ 1 h		56 (8.8)	37 (5.9)	
Participation in sports at age 4 years, yes	83 (6.3)	81 (13.1)	75 (12.1)	0.600
Nutritional intake at 4.5 years	95 (7.2)			
Total energy (kcal/day)		928.8 ± 360.7	857.2 ± 247.8	< 0.001
Carbohydrate (% energy)		57.4 ± 5.9	56.4 ± 5.6	0.002
Fat (% energy)		28.7 ± 5.0	29.6 ± 5.0	0.003
Protein (% energy)		12.4 ± 1.9	12.7 ± 1.7	0.022
Added sugar (% energy)		2.2 ± 2.1	2.1 ± 1.8	0.449

Here, *p* values were derived from chi-squared tests or Student’s *t*-tests. Data are presented as number (%) or mean ± standard deviation

^a^Either pre-existing or pregnancy-induced hypertension

^b^Either pre-existing or gestational diabetes

^c^Time spent playing with a mobile phone or electronic gaming console

We also assessed the predictive capability of adiposity timing for overweight or obesity at age 8 using the area under the receiver operating characteristic (ROC) curve. Analyses were limited to children with complete data. Overweight and obesity were defined using International Obesity Task Force age- and sex-specific BMI cutoffs, corresponding to BMI values of ≥ 25 and ≥ 30 at age 18, respectively [14]. Although waist circumference is commonly used in clinical and pediatric health screening in Japan to define central obesity—based on thresholds recommended in the Japanese pediatric metabolic syndrome screening guidelines (80 cm for children and adolescents and 75 cm for elementary-school-aged children) [21]—waist circumference was included in this study only as a supplementary descriptive indicator of adiposity. To account for multiple comparisons, we applied the Sidak correction, which assumes independence among tests and is slightly less conservative than the Bonferroni method while providing comparable control of the family-wise error rate. This approach was chosen because the conditional weight measures at each age point are statistically independent by construction. In our dataset, results were highly similar when using either the Sidak or Bonferroni correction, and the overall interpretation of statistical significance did not differ. Finally, to examine the potential mediating effect of concurrent obesity on the relationship between adiposity-related growth measures and ALT concentrations, models were further adjusted for BMI at age 8.

## Results

Of the 2120 eligible children, 798 were excluded due to missing blood ALT assessments or a history of infectious hepatitis (Fig. 1). Characteristics of included and excluded participants did not differ (Additional file 1: Table S2). At the 8-year follow-up, the median age was 96 months (IQR, 94–98 months) for both boys and girls. Median ALT concentration was 13 IU/L (IQR, 11–16 IU/L) in boys and 12 IU/L (IQR, 10–14 IU/L) in girls. Median AST and GGT concentrations were 26 IU/L (IQR, 24–29 IU/L) and 11 IU/L (IQR, 10–13 IU/L), respectively, in boys, and 25 IU/L (IQR, 23–28 IU/L) and 11 IU/L (IQR, 10–13 IU/L), respectively, in girls. Table 1 summarizes participant characteristics by child’s sex. Maternal sociodemographic and medical characteristics were generally comparable between boys and girls. However, higher proportions of boys than of girls experienced prolonged neonatal hospitalization and spent time playing with mobile phones or gaming consoles at age 3. Although energy intake from added sugar did not differ between sexes, boys tended to obtain a higher proportion of their daily energy intake from carbohydrates, whereas girls obtained more from fat and protein. During the first 3 years of life, girls’ weight and height aligned more closely with WHO growth standards than those of boys (Table 2). At age 8, 11.6% of boys and 10.8% of girls were classified as overweight or obese. Boys also exhibited significantly higher liver enzyme levels than girls at this age.

**Table 2:** Child anthropometry and ALT levels at age 8

Variables	Missing	Boys	Girls	*p*
	***n* (%)**	***n* = 665**	***n* = 657**	
**At birth**				
Gestational age at birth, wk	0	38.8 ± 1.4	38.8 ± 1.3	0.583
Weight z-score, SD	0	− 0.64 ± 0.8	− 0.62 ± 0.8	0.710
Length z-score, SD	5 (0.4)	− 0.47 ± 1.0	− 0.37 ± 1.1	0.086
**1 year**				
Age, mo	210 (15.9)	9.4 ± 1.5	9.5 ± 1.5	0.316
Weight z-score, SD	210 (15.9)	− 0.35 ± 0.9	− 0.19 ± 0.8	0.002
Height z-score, SD	213 (16.1)	− 0.67 ± 1.1	− 0.42 ± 0.9	< 0.001
Body mass index z-score, SD		0.06 ± 1.0	0.07 ± 0.9	0.856
Conditional weight	220 (16.6)	0.02 ± 0.7	− 0.000 ± 0.6	0.598
**2 years**				
Age, mo	95 (7.2)	22.3 ± 1.8	22.5 ± 1.6	0.032
Weight z-score, SD	100 (7.6)	− 0.38 ± 0.9	− 0.23 ± 0.8	0.001
Height z-score, SD	120 (9.1)	− 1.18 ± 1.0	− 1.07 ± 0.8	0.043
Body mass index z-score, SD		0.52 ± 1.0	0.58 ± 0.9	0.201
Conditional weight	303 (22.9)	0.004 ± 0.6	− 0.003 ± 0.5	0.824
**3 years**				
Age, mo	132 (10.0)	35.0 ± 1.6	35.1 ± 1.5	0.278
Weight z-score, SD	132 (10.0)	− 0.44 ± 0.9	− 0.33 ± 0.8	0.021
Height z-score, SD	155 (11.7)	− 1.01 ± 0.9	− 0.99 ± 0.8	0.721
Body mass index z-score, SD		0.27 ± 1.0	0.41 ± 0.9	0.013
Conditional weight	241 (18.2)	0.004 ± 0.4	0.02 ± 0.4	0.609
**4 years**				
Age, mo	108 (8.2)	46.9 ± 1.3	46.9 ± 1.5	0.826
Weight z-score, SD	111 (8.4)	− 0.45 ± 0.8	− 0.46 ± 0.7	0.880
Height z-score, SD	121 (9.2)	− 0.92 ± 0.9	− 0.97 ± 0.8	0.362
Body mass index z-score, SD		0.21 ± 0.9	0.20 ± 0.8	0.851
Conditional weight	228 (17.3)	0.02 ± 0.3	0.01 ± 0.3	0.607
**5 years**				
Age, mo	124 (9.4)	59.0 ± 1.4	58.9 ± 1.3	0.659
Weight z-score, SD	125 (9.5)	− 0.46 ± 0.8	− 0.48 ± 0.8	0.797
Height z-score, SD	130 (9.8)	− 0.80 ± 0.9	− 0.83 ± 0.8	0.563
Body mass index z-score, SD		0.06 ± 0.9	0.03 ± 0.8	0.470
Conditional weight	201 (15.2)	− 0.01 ± 0.4	− 0.01 ± 0.3	0.828
**6 years**				
Age, mo	124 (9.4)	70.7 ± 1.3	70.8 ± 1.3	0.427
Weight z-score, SD	124 (9.4)	− 0.39 ± 1.0	− 0.32 ± 0.8	0.134
Height z-score, SD	126 (9.5)	− 0.62 ± 0.9	− 0.55 ± 0.8	0.134
Body mass index z-score, SD		− 0.02 ± 1.0	0.000 ± 0.9	0.726
Conditional weight	200 (15.1)	0.005 ± 0.4	0.01 ± 0.3	0.739
**8 years**				
Age, mo	0	96.4 ± 3.4	96.1 ± 3.4	0.223
Weight z-score, SD	0	− 0.14 ± 1.2	− 0.13 ± 0.9	0.904
Height z-score, SD	0	− 0.32 ± 0.9	− 0.29 ± 0.8	0.475
Body mass index z-score, SD		0.04 ± 1.2	0.01 ± 0.9	0.578
Overweight or obesity		77 (11.6)	71 (10.8)	0.656
Waist circumference, cm	0	57.0 ± 6.4	56.2 ± 5.6	0.027
Waist-to-height ratio > 0.5		81 (12.2)	66 (10.1)	0.217
Conditional weight	130 (9.8)	− 0.01 ± 0.5	− 0.01 ± 0.4	0.983
**Liver enzymes at 8 years**				
log-ALT	0	2.61 ± 0.3	2.51 ± 0.3	< 0.001
Elevated ALT^a^		25 (3.8)	18 (2.7)	0.296
log-AST	0	3.28 ± 0.2	3.24 ± 0.2	< 0.001
log-GGT	1	2.43 ± 0.2	2.40 ± 0.2	0.030

Here, *p*-values were derived from Student’s t-tests or chi-squared tests

Data are presented as number (%) or mean ± standard deviation. Weight, length/height, and body mass index are expressed as z-scores transformed using WHO growth standards

Liver enzymes were expressed as log-transformed values

*ALT *alanine aminotransferase, *AST *aspartate aminotransferase, *GGT *γ-glutamyl transferase, *SD *standard deviation

^a^ALT elevation was defined as serum ALT > 26 IU/L in boys and > 22 IU/L in girls

Significant correlations between growth measures and ALT concentrations at age 8 were observed only with conditional weight measures (Additional file 1: Table S1), starting at 3 years in girls (*r* = 0.142; *p* < 0.05) and at 5 years in boys (*r* = 0.208; *p* < 0.001).

Figure 2 displays covariate-adjusted regression coefficients for the associations. Statistically significant associations with ALT concentrations were observed for conditional weight beginning at age 3 in girls (adjusted coefficients: 0.11, *p* < 0.01) and at age 4 in boys (0.10, *p* < 0.05). A noticeable increase in the magnitude of association emerged between ages 4 and 5, with adjusted coefficients of 0.19 (*p* < 0.001) in boys and 0.18 (*p* < 0.001) in girls. In contrast, associations between birth weight and ALT concentrations were inconsistent. Sensitivity analyses—including complete case analyses and those restricting the timing of anthropometric measurements—yielded similar results (Additional file 1: Fig. S1A, B, respectively). Unadjusted regression results for the primary, complete case, and restricted-timing analyses are provided in Additional file 1: Table S3.

Compared to children with average adiposity gain, those with excess gain at age 5 exhibited significantly higher risks of elevated ALT, with adjusted risk ratios (95% CI) of 4.18 (1.77–9.87) in boys and 3.29 (1.04–10.40) in girls (Table 3). Elevated risks were also observed in boys with excess conditional weight at ages 6 and 8. Complete case and restricted-timing analyses yielded consistent findings (Additional file 1: Table S4A, B).

**Table 3:** Association between excess adiposity and ALT elevation at age 8, stratified by child’s sex

**Adiposity gain**	**Boys**			**Girls**		
	**Elevated ALT**	**RR (95% CI)**	**aRR (95% CI)**	**Elevated ALT**	**RR (95% CI)**	**aRR (95% CI)**
**Between 2 and 3 years**						
Average	16 (3.4)	Ref	Ref	11 (2.3)	Ref	Ref
Excess	5 (8.1)	2.40 (0.91–6.33)	2.09 (0.77–5.70)	4 (7.1)*	3.16 (1.04–9.61)	2.72 (0.92–8.02)
**Between 3 and 4 years**						
Average	18 (3.8)	Ref	Ref	13 (2.6)	Ref	Ref
Excess	4 (6.6)	1.73 (0.60–4.95)	0.98 (0.37–2.60)	2 (3.6)	1.38 (0.32–5.96)	1.29 (0.29–5.81)
**Between 4 and 5 years**						
Average	15 (3.0)	Ref	Ref	12 (2.3)	Ref	Ref
Excess	8 (14.6)***	4.87 (2.16–10.97)	4.18 (1.77–9.87)	4 (8.0)*	3.43 (1.15–10.24)	3.29 (1.04–10.40)
**Between 5 and 6 years**						
Average	15 (3.1)	Ref	Ref	13 (2.5)	Ref	Ref
Excess	8 (11.8)**	3.83 (1.68–8.69)	2.66 (1.01–7.02)	2 (3.8)	1.49 (0.34–6.43)	1.27 (0.27–5.97)
**Between 6 and 8 years**						
Average	14 (2.6)	Ref	Ref	12 (2.2)	Ref	Ref
Excess	9 (14.1)***	5.34 (2.41–11.86)	5.79 (2.38–14.08)	3 (6.7)	3.06 (0.90–10.46)	2.32 (0.82–6.59)

*ALT *alanine aminotransferase, *RR *risk ratio, *aRR *risk ratio were adjusted for maternal pre-pregnancy body mass index, annual household income, maternal educational level, smoking and alcohol consumption during pregnancy, anemia during pregnancy, gestational age at birth (weeks), neonatal hospitalization, breastfeeding duration, child’s screen time at age 3 years, participation in sports at age 4 years, nutritional intake assessed at 4.5 years, and child’s age at ALT measurement (months). For the model evaluating adiposity gain at age 3 years, adjustments for screen time, participation in sports, and nutritional intake were not included. For the model evaluating adiposity gain at age 4 years, the adjustment for nutritional intake was not included

Significance levels (chi-squared tests): *p < 0.05, **p < 0.01, ***p < 0.001

Data are presented as number (%) or risk ratio (95% confidence interval). Excess adiposity was defined as conditional weight > 90th percentile of the population distribution. ALT elevation was defined as serum ALT level > 26 IU/L in boys and > 22 IU/L in girls

BMI at age 8 appeared to mediate a substantial proportion of the association between adiposity-related growth measures and ALT concentrations (Additional file 1: Fig. S2). Conditional weight from age 3 in girls and age 5 in boys showed statistically significant predictive ability for overweight or obesity at age 8 compared with birth weight (ROC analyses, Table 4). The interval between ages 4 and 5 represented the earliest period at which notable predictive ability for overweight or obesity at age 8 was observed, with area under the ROC curve of 0.808 for boys and 0.846 for girls (ROC analyses, Table 4).

**Table 4:** Prediction of overweight or obese at age 8 by timing of adiposity gain

Adiposity	Area under ROC curve (95% CI)
**Boys, *n* = 387**	
Birth weight z-scores (Reference)	0.522 (0.425–0.619)
Adiposity gain, birth–1y	0.617 (0.520–0.714)
Adiposity gain, 1–2y	0.594 (0.504–0.684)
Adiposity gain, 2–3y	0.704 (0.611–0.797)
Adiposity gain, 3–4y	0.680 (0.592–0.768)
Adiposity gain, 4–5y	0.808 (0.736–0.880)***
Adiposity gain, 5–6y	0.824 (0.753–0.894)***
Adiposity gain, 6–8y	0.811 (0.728–0.893)***
**Girls, *n* = 390**	
Birth weight z-scores (Reference)	0.523 (0.431–0.616)
Adiposity gain, birth–1y	0.637 (0.544–0.729)
Adiposity gain, 1–2y	0.607 (0.520–0.695)
Adiposity gain, 2–3y	0.711 (0.626–0.797)*
Adiposity gain, 3–4y	0.734 (0.653–0.814)**
Adiposity gain, 4–5y	0.846 (0.776–0.915)***
Adiposity gain, 5–6y	0.781 (0.699–0.863)**
Adiposity gain, 6–8y	0.859 (0.799–0.920)***

Adiposity gain reflects current weight adjusted for current height and weight at the preceding time point

Overweight and obesity were defined according to body mass index cutoffs recommended by the Childhood Obesity Working Group of the International Obesity Taskforce

Sidak-adjusted *p*-values: *p < 0.05, **p < 0.01, ***p < 0.001, compared with birth weight z-score

## Discussion

In this longitudinal cohort study of Japanese children, we observed that excess adiposity beginning at age 3—rather than during infancy—was significantly associated with subclinical ALT elevation at age 8. The associations between conditional weight and ALT levels were stronger and more consistent in boys than in girls. A noticeable increase in the magnitude of association with ALT and predictive ability for later obesity emerged between ages 4 and 5. In contrast, birth weight and conditional weight at ages 1 or 2 years showed weaker associations with subsequent obesity or elevated ALT, suggesting that associations between adiposity gain and obesity-related liver risk became more apparent during early childhood rather than infancy in this cohort.

These findings align with prior studies from Chile and Australia, which retrospectively tracked body weight and reported BMI divergences between children with and without MASLD beginning at ages 2 or 3 years [3, 5]. Using conditional weight—a metric adjusted for linear growth and prior weight—we extend these findings by providing a more refined estimate of adiposity gain and its metabolic consequences. Although adiposity gain between ages 4 and 5 marked the beginning of a stronger predictive trajectory for obesity at age 8 and a heightened association with ALT, it was not the peak period of risk. Rather, this stage may represent an early inflection point in the progression toward ectopic fat accumulation and metabolic programming.

Although intrauterine growth retardation has been proposed as a risk factor for pediatric MASLD [7, 8], we found no consistent associations between birth weight and ALT levels at age 8. Similarly, whereas infancy has been suggested as a sensitive period linking early adiposity to later hepatic outcomes [22], we observed no clear association between conditional weight at ages 1 or 2 and subsequent ALT levels. Developmental mismatch frameworks propose that rapid postnatal gain may be particularly detrimental among children with poor fetal growth [23], and several studies—often focusing on small-for-gestational-age infants—have reported that reduced fetal growth followed by rapid infant growth predicts adverse cardiometabolic outcomes [22, 24, 25].

In contrast to these early-life-focused findings, our results suggest that associations between adiposity gain and ALT emerge later, particularly between ages 3 and 5 years. This developmental stage coincides with adiposity rebound timing, when BMI reaches its nadir before rising again [26]. The timing of adiposity rebound is a well-established predictor of later obesity and metabolic risk, with early rebound—often occurring before age 5—consistently associated with higher BMI, insulin resistance, and other metabolic abnormalities in later life [27–30]. The emergence of ALT associations during this age window in our cohort may therefore reflect developmental changes around adiposity rebound that could increase susceptibility to adiposity-related liver alterations, although this cannot be determined from the present study.

Several factors may account for discrepancies across studies. Population-specific growth patterns, including variation in the timing of adiposity rebound, may influence when metabolic consequences of adiposity become detectable. In addition, hepatic vulnerability to ectopic fat accumulation and metabolic stress may strengthen as children transition from early to mid-childhood, making associations more evident during or shortly after adiposity rebound rather than during infancy [31]. Taken together, our findings suggest that although fetal and infant periods remain important for long-term metabolic programming, early childhood—particularly ages 3 to 5—may represent a developmental stage during which associations between adiposity gain, elevated ALT, and later cardiometabolic outcomes become detectable.

Sex-specific patterns also emerged. Boys exhibited higher ALT concentrations and stronger associations between adiposity gain and ALT levels than girls. Although baseline characteristics were generally similar, boys tended to obtain a higher proportion of their daily energy intake from carbohydrates, whereas girls obtained more from fat and protein. In addition, girls’ early childhood weight and height were more closely aligned with WHO growth standards. By age 8, boys had significantly greater waist circumference, likely reflecting differences in fat distribution. Visceral adipose tissue—an important contributor to hepatic free fatty acid flux and triglyceride synthesis [32]—is typically lower in girls, likely due to a more favorable visceral-to-subcutaneous fat ratio, a pattern that can emerge in early childhood [1, 2, 33]. Estrogen may confer further protection through its regulatory influence on lipid metabolism, insulin sensitivity, and inflammation [34]. Sex-specific susceptibility to early-life metabolic programming may also contribute to disparities in ALT levels and adiposity-related liver risk [18].

Overall, 3.3% of the study children exhibited ALT concentrations exceeding the biologically defined upper limit of normal. Although mild elevations can sometimes reflect physiological variation, they may also indicate underlying hepatic injury. MASLD is a common cause of ALT elevation in pediatric populations [11], and lifestyle interventions have shown effectiveness in improving MASLD [35, 36]. In this context, our findings indicate that excess adiposity gain after age 3 was associated with higher ALT concentrations at age 8, suggesting that early childhood may represent a developmental stage during which adiposity–ALT associations become more apparent. However, because ALT was assessed at a single time point, the temporal onset of liver enzyme alterations cannot be determined. These observations underscore the potential importance of monitoring weight trajectories beyond infancy, while acknowledging the need for further longitudinal investigation to clarify their clinical relevance.

## Strengths and limitations

A key strength of this study is the application of conditional growth modeling, allowing precise analysis of adiposity trajectories by accounting for prior growth. The ethnically homogeneous study population may reduce the risk of population-level confounding. In addition, longitudinal data and a relatively large sample enabled adjustment for multiple potential confounders, enhancing the reliability of the observed associations. Sensitivity analyses further supported the robustness of the findings. Moreover, age-specific cross-sectional analyses allowed examination of the ages at which associations between adiposity gain and later ALT alterations became observable, addressing the study’s primary aim of exploring potential time-specific patterns in these relationships. From a preventive perspective, complementary investigation of longitudinal patterns of weight gain may further elucidate how different growth trajectories relate to later liver outcomes. Nonetheless, residual confounding—particularly from unmeasured parental liver health, including undiagnosed MASLD—cannot be entirely excluded.

The primary limitation was the absence of serial ALT measurements, which precluded determination of when ALT elevations first developed and differentiation between transient and persistent elevations. Longitudinal assessment of liver markers is therefore warranted to determine whether the observed associations reflect temporary fluctuations or clinically meaningful hepatic injury. Moreover, data on alternative causes of elevated ALT—such as autoimmune hepatitis, alpha-1 antitrypsin deficiency, hemochromatosis, and celiac disease—were not available, limiting our ability to exclude other etiologies. However, given that the cohort largely comprises healthy, school-attending children, it is unlikely that participants had chronic major systemic or organ disorders affecting the observed association. Additional limitations include reduced participation related to disruptions caused by the COVID-19 pandemic and the fact that only 62.3% of eligible children underwent ALT assessment. Nevertheless, baseline characteristics did not significantly differ between children with and without ALT data, suggesting a low likelihood of selection bias. Variability in the timing of anthropometric assessments also represents a limitation; however, standardized age-adjusted measures were used to mitigate this heterogeneity. Although caregiver reports at age 6 did not identify any cases suggestive of precocious puberty among girls, the potential influence of pubertal status on ALT levels cannot be entirely excluded. Similarly, the lack of creatine kinase measurements precluded evaluation of potential muscular contributions to ALT levels. Nevertheless, the finding that concurrent obesity at age 8 substantially mediated the observed associations supports the interpretation that ALT variations were primarily attributable to adiposity-related factors. The low prevalence of elevated ALT resulted in wide confidence intervals in some models using a binary outcome, indicating reduced precision and possible underpowering; these results should therefore be interpreted cautiously. In contrast, analyses treating ALT as a continuous outcome—offering substantially greater statistical power—were less affected by this limitation. Finally, as the cohort was drawn exclusively from a single geographic area, regional factors, including local rates of childhood obesity, household structure, socioeconomic conditions, and access to nutritious foods, may have influenced the timing or magnitude of adiposity-related ALT elevations. Moreover, most children in this cohort had access to early childhood education settings, which may have influenced dietary and physical activity behaviors, potentially limiting generalizability of our findings to populations with different early-life environments.

## Conclusions

Our findings suggest that early childhood—particularly between ages 3 and 5 years—may represent a developmental period during which associations between adiposity gain and markers of liver health become observable in later childhood. Because ALT was assessed at a single time point, the timing of onset or progression of liver enzyme alterations cannot be determined, and causal inferences cannot be drawn from this observational study. Nevertheless, the observed patterns highlight the potential relevance of monitoring growth during this developmental stage. The sex-specific patterns observed in adiposity–ALT associations warrant further investigation using longitudinal assessments of liver markers and growth trajectories to clarify whether these differences reflect variation in timing, persistence, or developmental susceptibility.

## Supplementary Information

Additional file 1: Table S1. Pearson correlation coefficients between adiposity measures and log-transformed alanine aminotransferase levels at age 8. Table S2. Participant characteristics and childhood anthropometry by inclusion status. Table S3. Crude association between timing of adiposity gain and log-transformed alanine aminotransferase levels at age 8, stratified by sex. Table S4 (A). Association between excess adiposity and ALT elevation at age 8, stratified by child’s sex. Analysis includes 929 children with complete data on conditional weight at ages 3, 4, 5, 6, and 8 years. (B). Association between excess adiposity and ALT elevation in 8-year-old children, stratified by child’s sex. Analysis was restricted to children whose anthropometric measurements were obtained within ± 3 months of the target ages. Fig. S1. Association between timing of adiposity gain and log-transformed ALTlevels at age 8: (A). Analysis includes 929 children with complete data on conditional weight at ages 3, 4, 5, 6, and 8 years. (B). Analyses were restricted to children whose anthropometric measurements were obtained within ± 3 months of the target ages. Fig. S2. Association between timing of adiposity and log-transformed ALTlevels at age 8.

Additional file 2.

## Data Availability

Data are unsuitable for public deposition due to ethical restrictions and the legal framework of Japan. It is prohibited by the Act on the Protection of Personal Information (Act No. 57 of 30 May 2003, amended on 9 September 2015) to publicly deposit data containing personal information. Ethical Guidelines for Medical and Health Research Involving Human Subjects enforced by the Japan Ministry of Education, Culture, Sports, Science and Technology and the Ministry of Health, Labour and Welfare, also restricts the open sharing of the epidemiologic data. All inquiries about access to data should be sent to: [jecs-en@nies.go.jp] (mailto:jecs-en@nies.go.jp). The person responsible for handling enquires sent to this e-mail address is Dr. Shoji F. Nakayama, JECS Programme Office, National Institute for Environmental Studies.

## Abbreviations

ALT: Alanine aminotransferase
BMI: Body mass index
AST: Aspartate aminotransferase
COVID-19: Coronavirus disease 2019
GGT: γ-Glutamyl transferase
IQR: Interquartile range
JECS: The Japan Environment and Children’s Study
MASLD: Metabolic dysfunction-associated steatotic liver disease
ROC: Receiver operating characteristic curve
RR: Risk ratio
WHO: World Health Organization
