# Supplementary material for: Early childhood weight gain and alanine aminotransferase at age 8: an adjunct study of the Japan Environment and Children’s Study
Source: BMC Med. 2026 Mar 3;24:208. doi: 10.1186/s12916-026-04734-x (PMC13063647; doi:10.1186/s12916-026-04734-x)

Early childhood weight gain and alanine aminotransferase at age 8: An Adjunct Study of the Japan Environment and Children's Study

Additional file 1.

Table S1. Pearson correlation coefficients between adiposity measures and log-transformed alanine aminotransferase levels at age 8

Growth measure	Boys			Girls		
	Number	Mean ± SD	Pearson *r*	Number	Mean ± SD	Pearson *r*
Birth weight z-scores	665	–0.64 ± 0.8	–0.043	657	–0.62 ± 0.8	–0.017
Adiposity gain, birth–1y	562	0.02 ± 0.7	–0.026	540	–0.0001 ± 0.6	0.070
Adiposity gain, 1–2y	517	0.004 ± 0.6	0.015	502	–0.003 ± 0.5	0.081
Adiposity gain, 2–3y	538	0.004 ± 0.4	0.086	543	0.02 ± 0.4	0.142*
Adiposity gain, 3–4y	536	0.02 ± 0.3	0.098	558	0.01 ± 0.3	0.051
Adiposity gain, 4–5y	557	–0.01 ± 0.4	0.208***	564	–0.01 ± 0.3	0.171**
Adiposity gain, 5–6y	556	0.005 ± 0.4	0.184***	566	0.01 ± 0.3	0.105
Adiposity gain, 6–8y	596	–0.01 ± 0.5	0.312***	596	–0.01 ± 0.4	0.166**

Sidak-adjusted p-values: * $p < 0.05$, ** $p < 0.01$, *** $p < 0.001$.

Table S2. Participant characteristics and childhood anthropometry by inclusion status

Characteristics	Included<br>n = 1322	Excluded<br>n = 798	*p*
Maternal characteristics			
Age ≥35 years	385 (29.1)	221 (27.7)	0.481
Parity 1+	728 (57.9)	420 (53.8)	0.070
Marital status, married	1270 (98.5)	758 (97.3)	0.070
Pre-pregnancy body mass index			0.745
<18.5 kg/m^2	194 (14.7)	111 (13.9)	
18.5–24.9 kg/m^2	990 (74.9)	609 (76.4)	
≥25 kg/m^2	137 (10.4)	77 (9.7)	
Highest educational attainment, high school or less	317 (24.1)	197 (24.8)	0.734
Annual household income, <4 million Japanese Yen	503 (39.6)	304 (40.0)	0.872
Smoking status, non-smoker	874 (66.5)	542 (68.1)	0.455
Alcohol consumption			0.820
Never drink	424 (32.3)	253 (31.7)	
Quit during pregnancy	749 (57.0)	464 (58.2)	
Current drinking	141 (10.7)	80 (10.0)	
Presence of anemia	267 (20.3)	143 (17.9)	0.189
Presence of hypertension[a]	45 (3.4)	25 (3.1)	0.735
Presence of diabetes[b]	45 (3.4)	29 (3.6)	0.780
Child's characteristics			
Boys	665 (50.3)	406 (50.9)	0.798
Preterm birth	50 (3.8)	25 (3.1)	0.433
Neonatal hospital stays >7 days	307 (23.3)	216 (27.1)	0.049
Breastfeeding duration >6 months	1056 (79.9)	648 (81.2)	0.457
Daycare or kindergarten enrollment			0.031
Yes	1229 (93.0)	720 (90.2)	
No	12 (0.9)	16 (2.0)	
Not reported	81 (6.1)	62 (7.8)	
Screen time at age 3 years,[c] hours per day			0.634

0 h	708 (55.8)	404 (54.1)	
<1 h	467 (36.8)	281 (37.6)	
≥1 h	93 (7.3)	62 (8.3)	
Participation in sports at age 4 years, yes	156 (12.6)	71 (9.7)	0.047
Nutritional intake at 4.5 years			
Total energy (kcal/day)	893.0 ± 311.4	902.6 ± 260.3	0.487
Carbohydrate (% energy)	56.9 ± 5.8	57.0 ± 5.8	0.724
Fat (% energy)	29.2 ± 5.0	29.2 ± 5.0	0.913
Protein (% energy)	12.6 ± 1.8	12.4 ± 1.6	0.115
Added sugar (% energy)	2.2 ± 1.9	2.2 ± 1.9	0.875
**Child anthropometry**			
**At birth**			
Gestational age at birth, weeks	38.8 ± 1.3	38.9 ± 1.3	0.384
Weight z-score	–0.63 ± 0.8	–0.61 ± 0.8	0.700
Length z-score	–0.42 ± 1.0	–0.42 ± 1.0	0.922
**1 year**			
Weight z-score	–0.27 ± 0.9	–0.32 ± 0.9	0.243
Height z-score	–0.55 ± 1.0	–0.60 ± 1.0	0.339
Body mass index z-score	0.06 ± 0.9	0.04 ± 0.9	0.595
**2 years**			
Weight z-score	–0.31 ± 0.8	–0.32 ± 0.9	0.644
Height z-score	–1.13 ± 0.9	–1.09 ± 0.9	0.455
Body mass index z-score	0.55 ± 0.9	0.50 ± 0.9	0.241
**3 years**			
Weight z-score	–0.39 ± 0.8	–0.41 ± 0.8	0.502
Height z-score	–1.0 ± 0.9	–0.96 ± 0.9	0.362
Body mass index z-score	0.34 ± 0.9	0.28 ± 0.9	0.168
**4 years**			
Weight z-score	–0.46 ± 0.8	–0.51 ± 0.8	0.139
Height z-score	–0.94 ± 0.8	–0.91 ± 0.9	0.337
Body mass index z-score	0.21 ± 0.8	0.09 ± 0.8	0.004
**5 years**			

Weight z-score	–0.47 ± 0.8	–0.49 ± 0.8	0.686
Height z-score	–0.81 ± 0.8	–0.78 ± 0.9	0.466
Body mass index z-score	0.05 ± 0.9	0.001 ± 0.8	0.272
**6 years**			
Weight z-score	–0.35 ± 0.9	–0.37 ± 0.9	0.738
Height z-score	–0.59 ± 0.9	–0.57 ± 0.9	0.742
Body mass index z-score	–0.01 ± 0.9	–0.04 ± 0.9	0.503
**8 years**			
Weight z-score	–0.14 ± 1.1	–0.15 ± 1.1	0.850
Height z-score	–0.31 ± 0.8	–0.33 ± 0.9	0.591
Body mass index z-score	0.03 ± 1.1	0.04 ± 1.1	0.798
Waist circumference, cm	56.6 ± 6.0	57.1 ± 6.1	0.083

Data are presented as number (%) or mean ± standard deviation. The *p*-values were derived from chi-squared tests or Student's t-tests. Values for weight, length/height, and body mass index are presented as z-scores transformed using WHO standard.

[a]Either pre-existing or pregnancy-induced hypertension. [b]Either pre-existing or gestational diabetes. [c]Time spent playing with a mobile phone or electronic gaming consoles.

Table S3. Crude association between timing of adiposity gain and log-transformed alanine aminotransferase levels at age 8, stratified by sex.

Growth measures	Primary analyses[a]	Complete cases[b]	Restricted measurement timing[c]
**Boys**			
Birth weight z-scores	–0.02 (–0.05–0.01)	–0.02 (–0.06–0.02)	–0.02 (–0.05–0.01)
Adiposity gain, birth–1y	–0.01 (–0.05–0.03)	–0.04 (–0.09–0.01)	–0.02 (–0.06–0.02)
Adiposity gain, 1–2y	0.01 (–0.04–0.06)	–0.001 (–0.06–0.06)	0.01 (–0.04–0.07)
Adiposity gain, 2–3y	0.07 (0.001–0.14)	0.08 (–0.005–0.16)	0.10 (0.02–0.17)
Adiposity gain, 3–4y	0.09 (0.01–0.17)	0.08 (–0.02–0.17)	0.08 (–0.005–0.16)
Adiposity gain, 4–5y	0.19 (0.12–0.26)	0.18 (0.10–0.26)	0.15 (0.07–0.23)
Adiposity gain, 5–6y	0.15 (0.08–0.22)	0.18 (0.10–0.26)	0.17 (0.10–0.25)
Adiposity gain, 6–8y	0.19 (0.15–0.24)	0.20 (0.14–0.26)	0.20 (0.15–0.25)
**Girls**			
Birth weight z-scores	–0.01 (–0.03–0.02)	0.004 (–0.03–0.04)	–0.01 (–0.03–0.02)
Adiposity gain, birth–1y	0.03 (–0.01–0.07)	0.01 (–0.04–0.05)	0.03 (–0.01–0.08)
Adiposity gain, 1–2y	0.05 (–0.004–0.10)	0.05 (–0.01–0.11)	0.04 (–0.02–0.10)
Adiposity gain, 2–3y	0.11 (0.05–0.18)	0.11 (0.04–0.19)	0.10 (0.03–0.17)
Adiposity gain, 3–4y	0.05 (–0.03–0.12)	0.07 (–0.02–0.15)	0.05 (–0.03–0.13)
Adiposity gain, 4–5y	0.17 (0.09–0.25)	0.20 (0.11–0.29)	0.16 (0.08–0.25)
Adiposity gain, 5–6y	0.09 (0.02–0.17)	0.11 (0.02–0.19)	0.09 (0.02–0.17)
Adiposity gain, 6–8y	0.12 (0.06–0.17)	0.15 (0.09–0.22)	0.12 (0.06–0.18)

[a]Analyses includes all available data for each age period. [b]Analyses includes 929 children with complete data on conditional weight at ages 3, 4, 5, 6, and 8 years. [c]Analyses were restricted to children whose anthropometric measurements were obtained within ±3 months of the target ages. Values represent β coefficient (95% confidence interval) from linear regression models.

Table S4 (A). Association between excess adiposity and ALT elevation at age 8, stratified by child's sex. Analysis includes 929 children with complete data on conditional weight at ages 3, 4, 5, 6, and 8 years.

Adiposity gain	Boys			Girls		
	Elevated ALT	RR (95%CI)	aRR (95%CI)	Elevated ALT	RR (95%CI)	aRR (95%CI)
**Between 2 and 3 years**						
Average	16 (3.9)	Ref	Ref	10 (2.4)	Ref	Ref
Excess	5 (10.4)*	2.66 (1.02–6.95)	2.20 (0.80–6.07)	3 (6.1)	2.59 (0.74–9.10)	2.43 (0.67–8.86)
**Between 3 and 4 years**						
Average	17 (4.2)	Ref	Ref	11 (2.6)	Ref	Ref
Excess	4 (8.2)	1.96 (0.69–5.59)	1.11 (0.42–2.93)	2 (4.2)	1.61 (0.37–7.04)	1.42 (0.36–5.59)
**Between 4 and 5 years**						
Average	13 (3.1)	Ref	Ref	9 (2.1)	Ref	Ref
Excess	8 (19.1)***	6.08 (2.67–13.84)	4.95 (2.01–12.16)	4 (10.3)**	4.93 (1.59–15.31)	4.34 (1.15–16.39)
**Between 5 and 6 years**						
Average	14 (3.5)	Ref	Ref	11 (2.6)	Ref	Ref
Excess	7 (11.9)**	3.37 (1.42–8.02)	2.11 (0.79–5.63)	2 (4.4)	1.68 (0.38–7.38)	1.31 (0.27–6.30)
**Between 6 and 8 years**						
Average	14 (3.4)	Ref	Ref	10 (2.3)	Ref	Ref
Excess	7 (14.3)**	4.16 (1.76–9.82)	3.90 (1.56–9.78)	3 (7.9)*	3.43 (0.98–11.94)	2.23 (0.78–6.38)

Table S4 (B). Association between excess adiposity and ALT elevation in 8-year-old children, stratified by child's sex. Analysis was restricted to children whose anthropometric measurements were obtained within ±3 months of the target ages.

Adiposity gain	Boys			Girls		
	Elevated ALT	RR (95%CI)	aRR (95%CI)	Elevated ALT	RR (95%CI)	aRR (95%CI)
**Between 2 and 3 years**						
Average	13 (3.2)	Ref	Ref	10 (2.4)	Ref	Ref
Excess	5 (9.8)*	3.03 (1.13–8.16)	2.66 (0.97–7.25)	3 (6.1)	2.57 (0.73–9.04)	1.73 (0.62–4.86)
**Between 3 and 4 years**						
Average	16 (3.6)	Ref	Ref	13 (2.8)	Ref	Ref
Excess	3 (5.2)	1.45 (0.43–4.83)	1.15 (0.43–3.09)	2 (3.7)	1.34 (0.31–5.81)	1.27 (0.28–5.63)
**Between 4 and 5 years**						
Average	14 (3.0)	Ref	Ref	11 (2.3)	Ref	Ref
Excess	7 (14.3)***	4.77 (2.02–11.25)	4.83 (2.08–11.24)	5 (10.6)**	4.65 (1.69–12.83)	4.44 (1.57–12.56)
**Between 5 and 6 years**						
Average	15 (3.3)	Ref	Ref	12 (2.5)	Ref	Ref
Excess	8 (12.7)**	3.90 (1.72–8.84)	2.69 (1.00–7.23)	2 (4.0)	1.63 (0.38–7.10)	1.47 (0.29–7.49)
**Between 6 and 8 years**						
Average	14 (2.7)	Ref	Ref	11 (2.1)	Ref	Ref
Excess	9 (14.5)***	5.32 (2.40–11.79)	5.86 (2.42–14.18)	3 (6.8)*	3.32 (0.96–11.46)	2.89 (1.07–7.82)

ALT, alanine aminotransferase; RR, risk ratio; aRR, risk ratio were adjusted for maternal pre-pregnancy BMI, annual household income, maternal educational level, smoking and alcohol consumption during pregnancy, anemia during pregnancy, gestational age at birth (weeks), neonatal hospitalization, breastfeeding duration, child's screen time at age 3 years, participation in sports at age 4 years, nutritional intake assessed at 4.5 years, and child's age at ALT measurement (months). For the model evaluating adiposity gain at age

3 years, adjustments for screen time, participation in sports, and nutritional intake were not included. For the model evaluating adiposity gain at age 4 years, the adjustment for nutritional intake was not included.

Significance levels (chi-squared tests): * $p < 0.05$, ** $p < 0.01$, *** $p < 0.001$.

Data are presented as number (%) or risk ratio (95% confidence interval). Excess adiposity was defined as conditional weight >90th percentile of the population distribution. ALT elevation was defined as serum ALT level >26 IU/L in boys and >22 IU/L in girls.

Fig. S1. Association between timing of adiposity gain and log-transformed ALT (log-ALT) levels at age 8: (A) Analysis includes 929 children with complete data on conditional weight at ages 3, 4, 5, 6, and 8 years. **(B)** Analyses were restricted to children whose anthropometric measurements were obtained within ±3 months of the target ages. Values represent regression coefficients (95% confidence intervals) and corresponding *p*-values from linear regression models. All models were adjusted for maternal pre-pregnancy BMI, annual household income, educational level, smoking and alcohol consumption during pregnancy, anemia during pregnancy, gestational age at birth (weeks), and the child's age at ALT measurement (months). For models evaluating adiposity gain up to 3 years, additionally adjusted for neonatal hospitalization and breastfeeding duration. For models assessing adiposity gain at age 4 years, additional adjustments included participation in sports and screen time. For models assessing adiposity gain at age 5 and later, we further adjusted for nutritional intake at 4.5 years. ALT, Alanine aminotransferase.

(A)

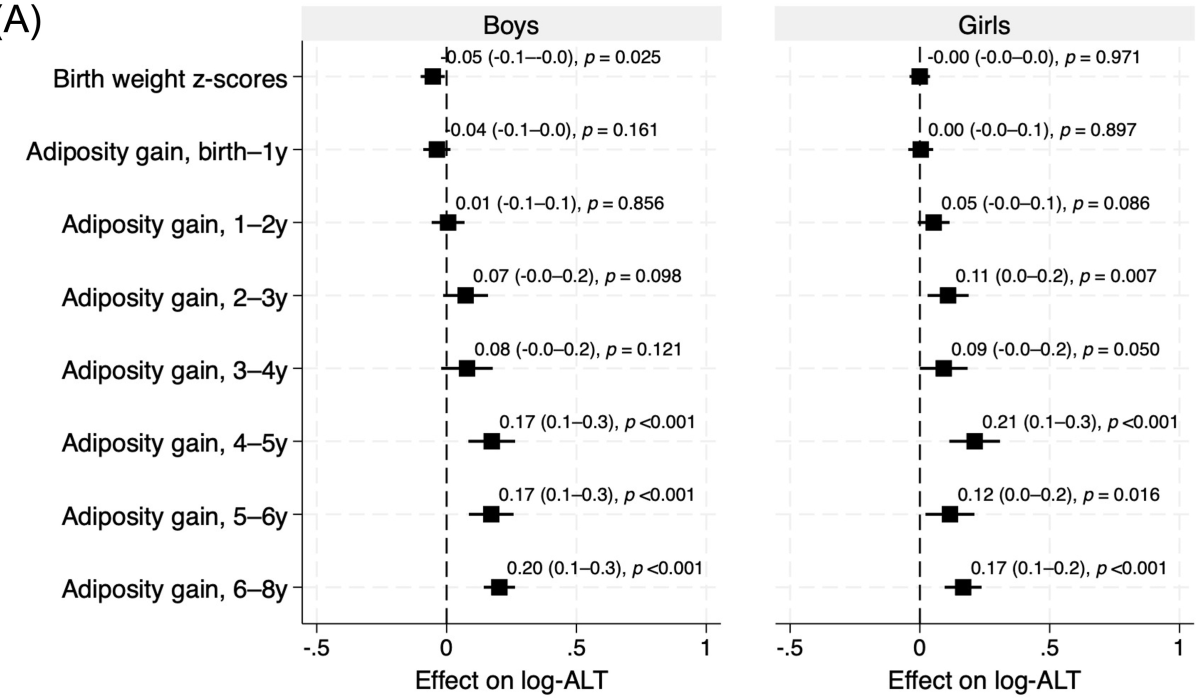

(B)

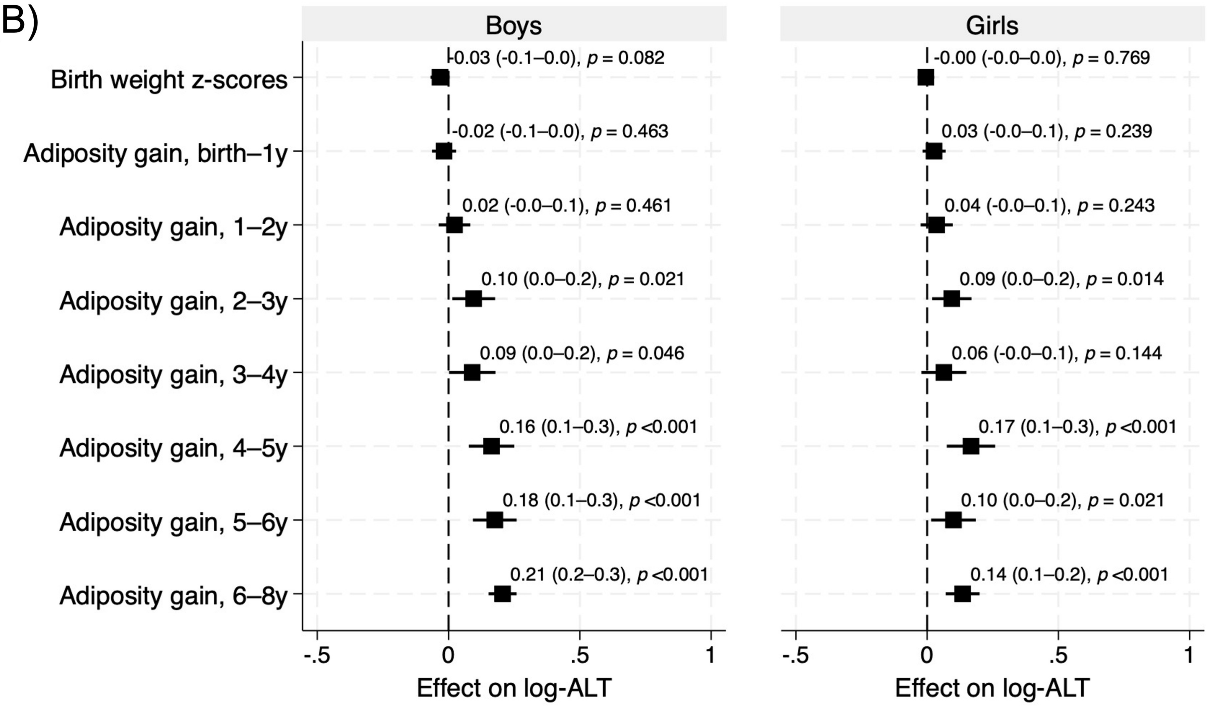
Boys
Girls
Birth weight z-scores
Adiposity gain, birth–1y
Adiposity gain, 1–2y
Adiposity gain, 2–3y
Adiposity gain, 3–4y
Adiposity gain, 4–5y
Adiposity gain, 5–6y
Adiposity gain, 6–8y
-0.03 (-0.1–0.0), p = 0.082
-0.02 (-0.1–0.0), p = 0.463
0.02 (-0.0–0.1), p = 0.461
0.10 (0.0–0.2), p = 0.021
0.09 (0.0–0.2), p = 0.046
0.16 (0.1–0.3), p <0.001
0.18 (0.1–0.3), p <0.001
0.21 (0.2–0.3), p <0.001
-0.00 (-0.0–0.0), p = 0.769
0.03 (-0.0–0.1), p = 0.239
0.04 (-0.0–0.1), p = 0.243
0.09 (0.0–0.2), p = 0.014
0.06 (-0.0–0.1), p = 0.144
0.17 (0.1–0.3), p <0.001
0.10 (0.0–0.2), p = 0.021
0.14 (0.1–0.2), p <0.001
-.5
0
.5
1
Effect on log-ALT

Fig. S2. Association between timing of adiposity and log-transformed ALT (log-ALT) levels at age 8. Models were adjusted for the child's body mass index (BMI) at age 8 to assess the mediating role of concurrent obesity in the association between adiposity-related measures and ALT concentrations. Values represent regression coefficients (95% confidence intervals) and corresponding *p*-values from linear regression models. All models were adjusted for maternal pre-pregnancy BMI, annual household income, educational level, smoking and alcohol consumption during pregnancy, anemia during pregnancy, gestational age at birth (weeks), the child's age at ALT measurement (months), and child's BMI at age 8. For models evaluating adiposity gain up to 3 years, additionally adjusted for neonatal hospitalization and breastfeeding duration. For models assessing adiposity gain at age 4 years, additional adjustments included participation in sports and screen time. For models assessing adiposity gain at age 5 and later, we further adjusted for nutritional intake at 4.5 years. ALT, Alanine aminotransferase.

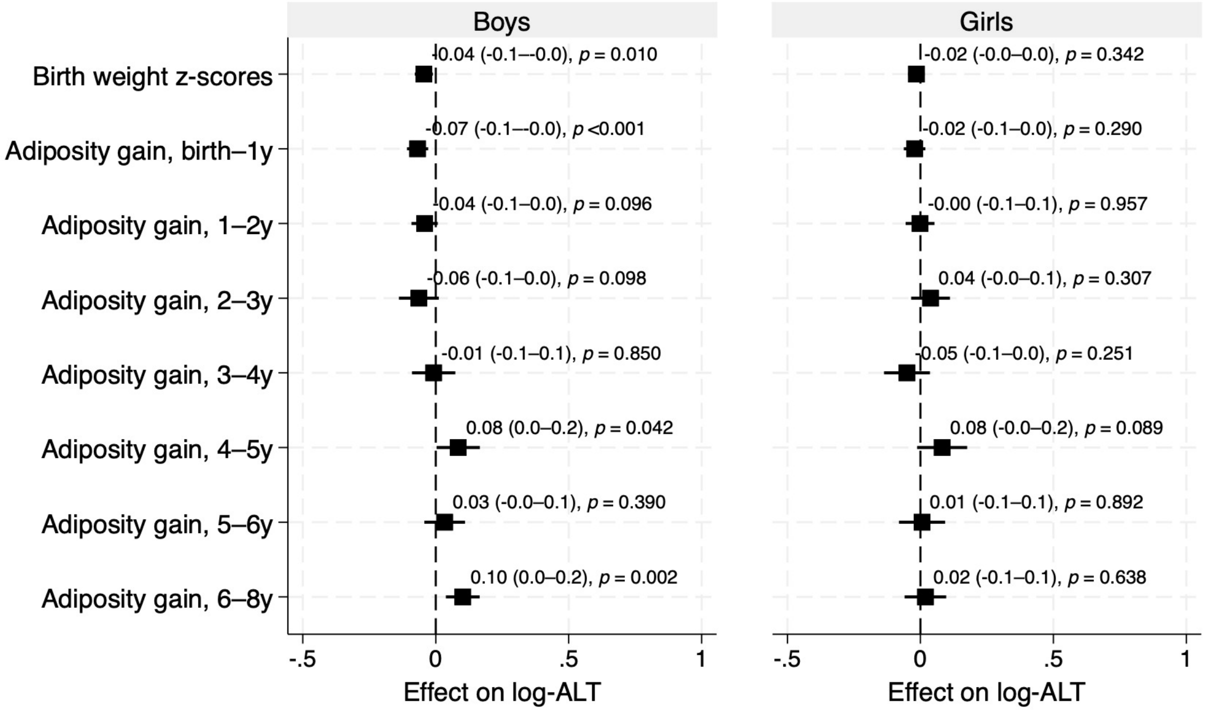

Supplement: Supplementary file 1 — Additional file 1: Table S1. Pearson correlation coefficients between adiposity measures and log-transformed alanine aminotransferase levels at age 8. Table S2. Participant characteristics and childhood anthropometry by inclusion status. Table S3. Crude association between timing of adiposity gain and log-transformed alanine aminotransferase levels at age 8, stratified by sex. Table S4 (A). Association between excess adiposity and ALT elevation at age 8, stratified by child’s sex. Analysis includes 929 children with complete data on conditional weight at ages 3, 4, 5, 6, and 8 years. (B). Association between excess adiposity and ALT elevation in 8-year-old children, stratified by child’s sex. Analysis was restricted to children whose anthropometric measurements were obtained within ± 3 months of the target ages. Fig. S1. Association between timing of adiposity gain and log-transformed ALTlevels at age 8: (A). Analysis includes 929 children with complete data on conditional weight at ages 3, 4, 5, 6, and 8 years. (B). Analyses were restricted to children whose anthropometric measurements were obtained within ± 3 months of the target ages. Fig. S2. Association between timing of adiposity and log-transformed ALTlevels at age 8. [file 12916_2026_4734_MOESM1_ESM.pdf]
